# Spectral Reflectance Indexes Reveal Differences in the Physiological Status of *Brassica oleracea* with Contrasting Glucosinolate Content under Biotic Stress

**DOI:** 10.3390/plants12142698

**Published:** 2023-07-19

**Authors:** Pilar Soengas, Pari Madloo, Margarita Lema

**Affiliations:** Group of Genetics, Breeding and Biochemistry of Brassicas, Misión Biológica de Galicia, Spanish Council for Scientific Research (MBG-CSIC), 36143 Pontevedra, Spain; paribroukani@gmail.com (P.M.); mlema@mbg.csic.es (M.L.)

**Keywords:** plant secondary metabolites, *Xanthomonas campestris* pv. *campestris*, *Sclerotinia sclerotiorum*, isothiocyanates, growth–immunity tradeoff

## Abstract

*Brassica* species produce glucosinolates, a specific group of secondary metabolites present in the Brassicaceae family with antibacterial and antifungal properties. The employment of improved varieties for specific glucosinolates would reduce the production losses caused by pathogen attack. However, the consequences of the increment in these secondary metabolites in the plant are unknown. In this work, we utilized reflectance indexes to test how the physiological status of *Brasica oleracea* plants changes depending on their constitutive content of glucosinolates under nonstressful conditions and under the attack of the bacteria *Xanthomonas campestris* pv. *campestris* and the fungus *Sclerotinia sclerotiorum*. The modification in the content of glucosinolates had consequences in the resistance to both necrotrophic pathogens, and in several physiological aspects of the plants. By increasing the content in sinigrin and glucobrassicin, plants decrease photosynthesis efficiency (PR531, F_v_F_m_), biomass production (CHL-NDVI, SR), pigment content (SIPI, NPQI, RE), and senescence (YI) and increase their water content (WI900). These variables may have a negative impact in the productivity of crops in an agricultural environment. However, when plants are subjected to the attack of both necrotrophic pathogens, an increment of sinigrin and glucobrassicin confers an adaptative advantage to the plants, which compensates for the decay of physiological parameters.

## 1. Introduction

Glucosinolates (GSLs) are secondary metabolites found in 16 botanical families of dicotyledonous angiosperms, mainly of the order of Brassicales, and are particularly predominant in the Brassicaceae family [1]. They are derived from aminoacids and they can be classified into three chemical classes, depending on the side chain of their precursor amino acid: aliphatics with methionine, alanine, valine, leucine, and isoleucine as precursors; aromatics derived from phenylalanine or tyrosine and indolics with tryptophan as a precursor [2].

GSLs are stored in plant cells in a non-activated form. Upon cellular disruption caused by injuries, pests, and necrotrophic pathogens, myrosinase enzymes come into contact with GSLs and catalyze their hydrolysis into various hydrolytic products, which have antimicrobial effect against a broad range of plant pathogens. The effect of hydrolitic products depends on their chemical structure and on the plant pathogen [3,4,5,6]. The GSLs-myrosinase system is part of the immune system of Brassicaceae plants, including *Arabidopsis thaliana* and *Brassica* crops [7,8,9,10]. GSLs are constitutively synthetized and stored in plant cells and act mainly as phytoanticipins, although they can be induced by pathogen infection functioning in this case, as phytoalexins [3,4]. 

Previously, we used mass selection to develop a set of *Brassica oleracea* var. *acephala* L. (kale) populations differing in the content of two GSLs, the aliphatics sinigrin (2-propenyl, SIN) and the indolic glucobrassicin (3-indolylmethyl, GBS) [11], obtaining four populations, with high (HSIN and HGBS) and low content (LSIN and LGBS) of the target GSLs. The resistance of these four populations have been previously tested against two *Brassica* pathogens: the fungus *Sclerotinia sclerotiorum* (*Ss*) and the bacteria *Xanthomonas campestris* pv. *campestris* (*Xcc*) [8]. Results showed that the effects of GSLs were dependent on the pathogen and the type of GSL. Thus, the aliphatic SIN was inhibitory to infection by *Ss* and the indolic GBS was inhibitory to infection by *Xcc* [8]. 

The employment of improved varieties for specific GSLs would reduce the yield and economic losses caused by the attack of pathogens. However, we do not know how the selection affects other parameters in the plant and its potential impact on productivity. Although high constitutive content of defensive metabolites may confer an advantage to plants subjected to pathogen attack, their synthesis is resource-demanding and may in turn become a disadvantage under non-stressful conditions [12]. Constitutive defense responses may reduce the growth of plants due in part to the unnecessary diversion of energy reserves in the absence of stress [12]. Increased expression of defense traits consumes metabolic resources at the cost of growth. For example, the Arabidopsis R-gene RPM1 costs 9% of the yield when plants are not under attack from the pathogen to which this gene confers resistance [13]. Moreover, growth and defense are negatively regulated not only through metabolic consumption but also through the antagonism of defensive phytohormones, such as jasmonic acid and salicylic acid [14].

Theoretically, GSL production can increase photosynthetic requirements in *A. thaliana* by at least 15% [15]. This cost associated with the production of GSLs may impact other processes of the plant and affect fitness and growth. Defense-associated costs may be particularly important in crops of the same family, since it can reduce their production and economic value. The study of the balance between growth and defense should be considered before releasing new improved varieties with enhanced GSL content. 

Plant reflectance is influenced by leaf surface properties and internal structure, as well as by the concentration and distribution of biochemical components, such as chlorophyll and carotenoid pigments. Therefore, analysis of reflectance can be used to assess both the biomass and the physiological status of a plant [16]. In this work, we utilized reflectance measurements to test how the physiological status of *B. oleracea* plants changes depending on their constitutive content of GSLs under non-stressful conditions and when challenged by pathogen attack. We also discuss the concerns in breeding for resistance to pathogens. 

## 2. Results

Leaf reflectance was measured and recorded in plants with contrasting content of GSLs under control conditions and inoculated with *Xcc* and *Ss* pathogens at different times post inoculation. Resulting profiles revealed specific spectral patterns for populations and treatments (Supplementary Figures S1–S4). Thus, we computed indexes based on reflectance to compare the performance of plants differing in GSL content and to compare between treatments. The results of each comparison are shown below. 

**Figure 1:**
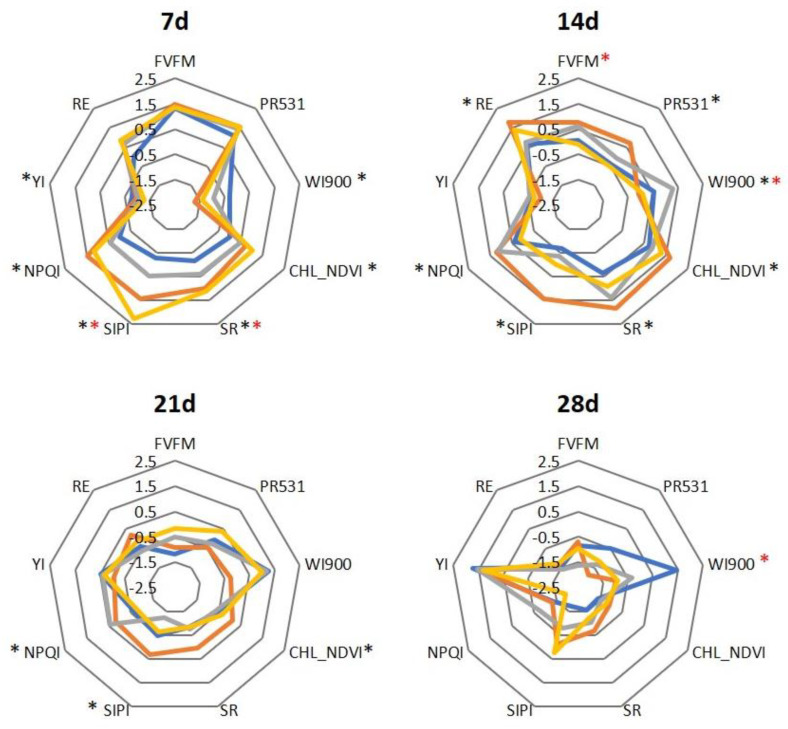
Spider plots showing the averaged reflectance indexes and fluorescence of control plants during four weeks in two populations differing in their content of GBS (HGBS and LGBS) and in two populations differing in their content of SIN (HSIN and LSIN). To represent the indexes in the same scale, values were standardized against time by subtracting the average and dividing by the standard error. Asterisks represent significant differences between HGBS and LGBS (black) and between HSIN and LSIN (red) at *p* ≤ 0.05.

Generally speaking, the population HGBS showed lower index values than LGBS against time, although differences were not always significant (Figure 1). There were not many significant differences between HSIN and LSIN (Figure 1), although, as it happens with GBS populations, HSIN trended to have lower values in the indexes than LSIN. The exception is the index WI900, which was higher in HGBS and HSIN than in LGBS and LSIN, respectively (Figure 1). Plants decrease their values in the different indexes against time, with the exception of YI and SIPI (Figure 1), related to the yellowing and senescence of leaves, which increases against time. 

**Figure 2:**
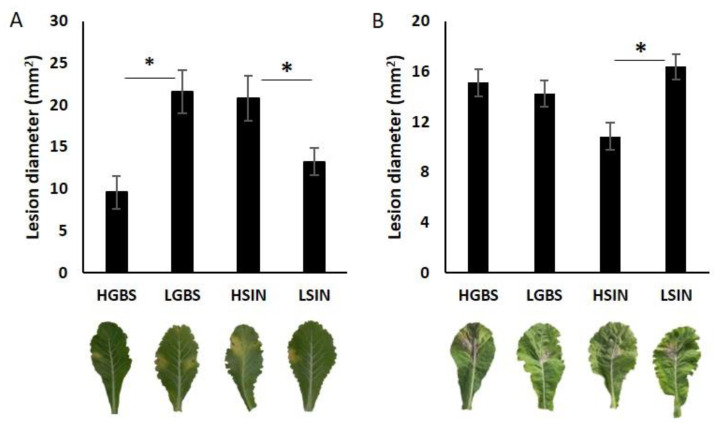
Lesion diameter of inoculated plants: (**A**) *Xanthomonas campestris* pv. *campestris* measured 21 days post inoculation; (**B**) *Sclerotinia sclerotiorum* measured 4 days post inoculation. Asterisks represent significant differences between populations at *p* ≤ 0.05.

Comparisons between control and inoculated plants are shown at the time when infection reached its maximum level: 21 days post inoculation in *Xcc* experiment and 4 days post inoculation in *Ss* experiment. In the *Xcc* experiment, HGBS showed less damage than LGBS and LSIN showed less damage than HSIN (Figure 2A). In the *Ss* experiment, HSIN was significantly less damaged than LSIN (Figure 2B).

**Figure 3:**
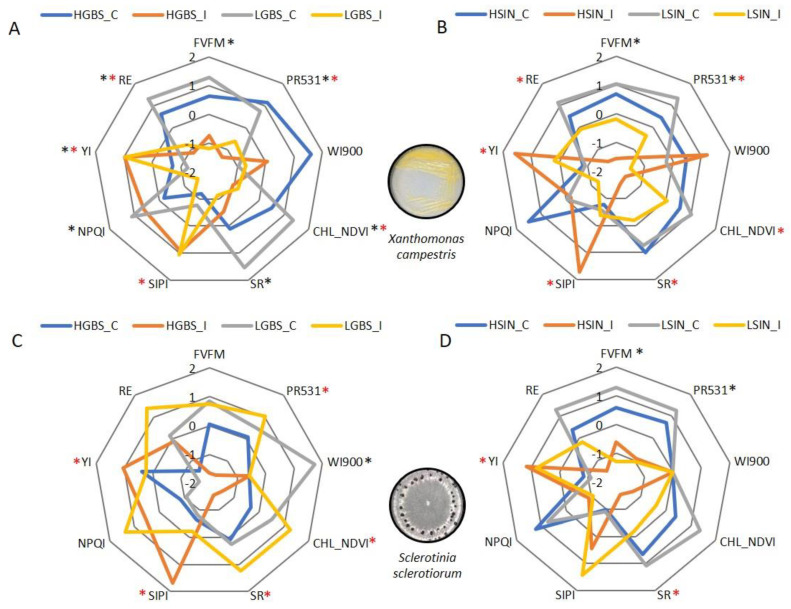
Spider plots showing the averaged reflectance indexes and fluorescence of controls and inoculated plants of two populations differing in their content of GBS (HGBS and LGBS) and SIN (HSIN and LSIN) with pathogens *Xanthomonas campestris* pv. *campestris* after 21 days post inoculation (**A**,**B**) and *Sclerotinia sclerotiorum* after 4 days post inoculation (**C**,**D**). To represent the indexes in the same scale, values were standardized against time by subtracting the average and dividing by the standard error. Asterisks represent significant differences between the high GSL content population and its respective control (black) and between the low GSL content population and its respective control (red) at *p* ≤ 0.05.

Controls (non-inoculated leaves) of HGBS and LGBS populations showed higher values of indexes F_v_F_m_, PR531, CHL-NDVI, SR, and RE than the respective inoculated populations with *Xcc* and lower values of SIPI and YI, although these differences were not always significant (Figure 3A). A similar trend was observed when we compare HSIN and LSIN controls with inoculated populations with *Xcc*, although significant differences were found mainly in LSIN comparisons (Figure 3B). 

In the *Ss* experiment, HGBS control only differed significantly from inoculated plants for index WI900 (Figure 3C). Control of LGBS showed significantly lower values of PR531, CHL-NDVI, SR, SIPI, and YI than inoculated plants. With respect to HSIN and LSIN populations, indexes related to biomass and photosynthesis are higher in control plants compared to inoculated ones, although differences were only significant for F_v_F_m_ and PR531 in HSIN and RE in LSIN. Those indexes related to the degradation of pigments and yellowness tended to be higher in inoculated plants, although differences were only significant for YI in LSIN (Figure 3D).

**Figure 4:**
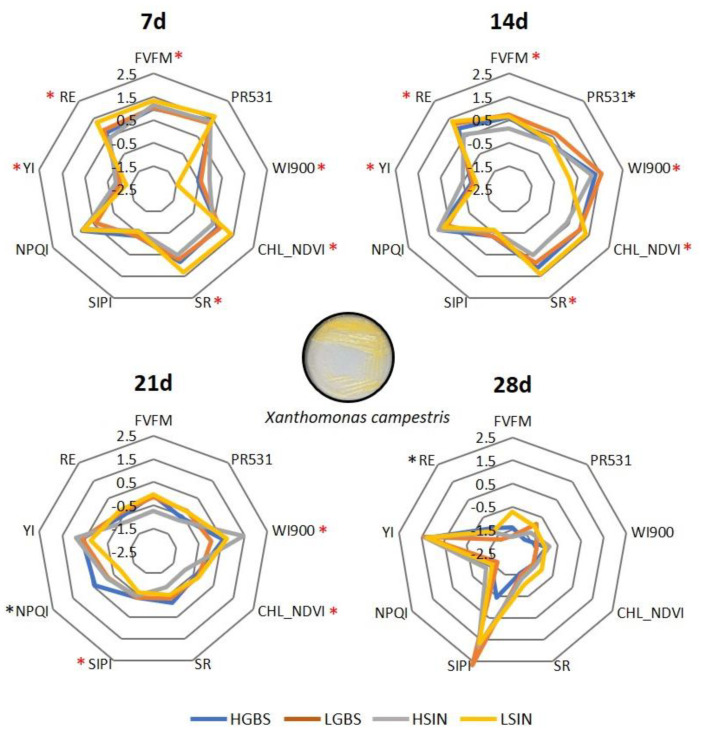
Spider plots showing the averaged reflectance indexes and fluorescence of inoculated plants with *Xanthomonas campestris* pv. *campestris* during four weeks in two populations differing in their content of GBS (HGBS and LGBS) and in two populations differing in their content of SIN (HSIN and LSIN). To represent the indexes in the same scale, values were standardized against time by subtracting the average and dividing by the standard error. Asterisks represent significant differences between HGBS and LGBS (black) and between HSIN and LSIN (red) at *p* ≤ 0.05.

In the comparison of populations with high and low content of GSLs after being challenged with pathogens, we found that there are not many significant differences between HGBS and LGBS against time after being inoculated with *Xcc*, on the contrary to what happened with the controls (Figure 4). LSIN showed, in general, higher values in the indexes at 7 and 14 d post inoculation than HSIN when both populations were inoculated with *Xcc*, with the exception of the index YI, which was higher in HSIN than in LSIN (Figure 4).

**Figure 5:**
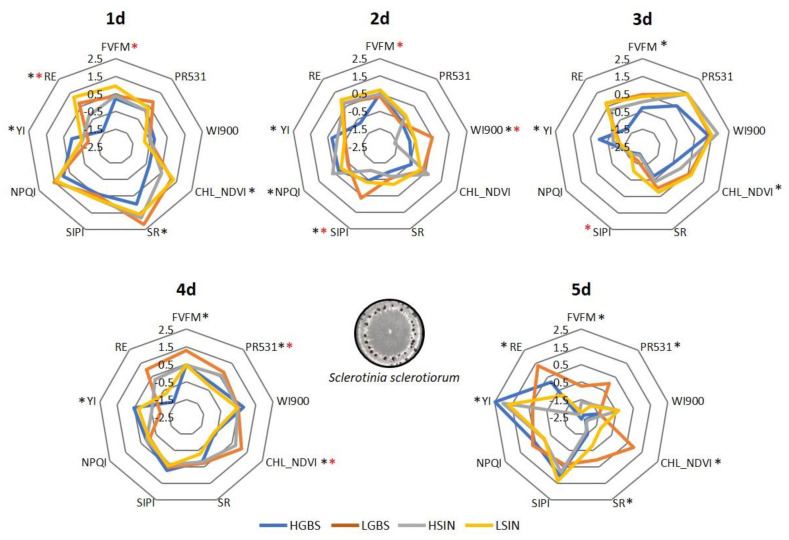
Spider plots showing the averaged reflectance indexes and fluorescence of inoculated plants with *Sclerotinia sclerotiorum* during five days post inoculation in two populations differing in their content of GBS (HGBS and LGBS) and in two populations differing in their content of SIN (HSIN and LSIN). To represent the indexes in the same scale, values were standardized against time by subtracting the average and dividing by the standard error. Asterisks represent significant differences between HGBS and LGBS (black) and between HSIN and LSIN (red) at *p* ≤ 0.05.

When HGBS and LGBS were inoculated with *Ss*, LGBS showed higher values for most of the indexes against time, except YI, which was consistently and significantly higher in HGBS (Figure 5). At 4 d post inoculation, HSIN showed higher values of PR531, CHL550, and CHL-NDVI than LSIN (Figure 5). 

## 3. Discussion

Leaf reflectance is a complex phenomenon dependent on biochemical and biophysical properties of the canopy leaves, which in turn are affected by growth conditions and diseases [17]. Photosynthetic pigments (chlorophylls and carotenoids) absorb light in the visible spectrum (400–700 nm). In the near infrared domain (700–1300 nm) reflectance is influenced by the structure of the leaf and water content, while in the middle infrared region (1300–3000 nm) the variability of reflectance is linked to water content and the composition of leaf chemicals [16]. 

In our experiments we have found that high and low GSL content populations differed in their spectral patterns. Following [18], the absorbance at 425 nm is correlated to total GSL content in *Brassica napus*, while [19] found that the spectral reflectance in several bands between 742 and 1000 nm is strongly correlated to individual GSL content in kale (*B. oleracea*). Other differences in the spectral reflectance between populations with contrasting GSL content reflect differences in their vigor, photosynthesis, or water content. We will discuss in the next section how differences in the constitutive content of GSLs and the application of biotic stressors modulate indexes based on reflectance in the infrared region of the spectrum and its relationship to the overall physiological status of the plants. 

Based on reflectance indexes and in the F_v_/F_m_, LGBS has more biomass, it is more photosynthetically efficient, it has less water content, and less degradation of pigments than HGBS. The same trend is observed when we compare LSIN and HSIN. Therefore, modification of GSL content affects plant physiological status. GSLs are part of the defense system of Brassicaeae plants. However, they can also interfere with other processes in the plant, although mechanisms governing this interaction are not clear. Some evidences suggest that there is a cross-talk of the biosynthetic GSL pathway with the hormone metabolism, stomatal aperture, the circadian clock, root growth, biomass, and flowering [20,21,22,23,24,25,26,27].

In this work, HGBS and HSIN showed higher values in the water index compared to LGBS and LSIN during the first 14 days of the experiment. Probably, these differences are related to the stomatal closure promoted by a higher content in GSLs. Reference [27] found that *tgg1* mutants, deficient in the myrosinase that catalyzes the production of isothiocyanates (ITCs) from GSLs, were hyposensitive to abscisic acid (ABA) inhibition of guard cell inward K^+^ channels and stomatal opening, revealing that the GSL–myrosinase system is required for key ABA responses of guard cells. Moreover, the addition of SIN in *B. oleracea* exposed to salinity stress can regulate aquaporins and water transport [25]. GSLs themselves do not suffice to inhibit channel activity, suggesting that it is the hydrolyzed products of the same that evoke ion channel inhibition [27]. Agreeing with this, the exogenous application of allyl-ITC, a degradation product from SIN, induced stomatal closure in *Arabidopsis thaliana* via the production of reactive oxygen species, nitric oxide, and an increase in cytosolic Ca^2+^ [28]. 

Moreover, the interaction of the GSL–myrosinase system with ABA signaling could also modify the senescence of leaves, since ABA positively regulates this process [29]. Increases in the relative concentration of carotenoids with respect to chlorophyll are often observed when plants are subjected to stress and in senescing leaves [16]. Indexes YI and SIPI are based on this assumption. In our experiments, they increase with the age of the leaf as is expected and decrease in increments in the constitutive content of GSLs. Therefore, high GSLs content would promote an increase in water content and delay in senescence.

Another remarkable result from our experiments is that increments in the GBS content in leaves lead to a decrease in photosynthesis and biomass accumulation. It is possible that this effect is driven by the hydrolytic products of the GSL. The biomass of *A. thaliana* roots decreases after the external addition of indol-3-carbinol, the main hydrolytic product from GBS, in the leaves by inhibiting auxin signaling through binding to the *tir1* auxin receptor [23,30]. On the contrary, [31] evaluated the agronomic performance of HGBS and LGBS in different environments and analyzed their metabolomic profile, finding that HGBS had a significant increase in fresh and dry foliar weight compared to LGBS. Reference [31] hypothesized that the better agronomic performance of HGBS could be related to an indirect increase in flavonoids which can act as growth regulators. Our experiments were carried out with seedlings, under greenhouse conditions, whereas the experiments of [31] were conducted in field conditions with adult plants exposed to the attack of pests, pathogens, and abiotic stresses. Therefore, it is possible that, in field conditions, plants from HGBS have an advantage over those from LGBS, which have to induce the synthesis of more secondary metabolites and, thus, relocate resources from the primary metabolism. 

Plants from the HSIN population also tend to have lower photosynthesis and biomass indexes than LSIN. Reference [20] found that exogenous SIN application lead to variations in biomass in *A. thaliana* that were dependent on the sugar concentration and on the endogenous GSL content of the plant. In this way, plant biomass accumulation was negatively correlated with the ratio of methylthioalkyl/methylsulfinylalkyl GSL and positively correlated with total aliphatic GSL accumulation [20]. The effect of SIN on biomass may be caused by its hydrolysis by myrosinases, since the addition of allyl-ITC caused growth inhibition in *A. thaliana* [26]. As was mentioned before, ITC treatments lead to stomatal closure and this could also be related to the loss of biomass. 

In summary, increments in the constitutive content of individual GSLs lead to a decrease in photosynthesis efficiency, biomass production, and senescence and to an increment in water content. This effect was independent of the chemical class of GSLs, although the responses were stronger in GBS genotypes. The physiological effects of GSLs may be driven by their hydrolytic products, which could take place after puncturing the leaves in control plants. The relationship of the amount of GSLs with the physiological status of the plant may respond to a growth–defense tradeoff, where resources are sent to the immune system rather than to growth.

To test this hypothesis, we challenged the four genotypes with necrotrophic pathogens. We found differences in the spectral reflectance between control and inoculated plants in both experiments (Supplementary Figures S1–S4). The physiological performance of populations is compromised by their interaction with surrounding pathogens. In fact, indexes based on reflectance have been employed to study the infection caused by *Xanthomonas* species in tangerine [32] and rice [33] plants. Spectral indexes were effective in the early detection of *X. campestris* in broccoli plants under different climatic conditions [17]. In addition, spectral characteristics (ranging from 1000 to 2500 nm) were successfully used to discriminate leaves inoculated with *Alternaria dauci* from the other three species of *Alternaria* as well as from control plants [34]. As expected, the controls showed higher indexes of photosynthesis and biomass, and lower indexes of senescence than their respective inoculated populations. Surprisingly, the LGBS population does not show this trend when inoculated with *Ss*. 

Differences observed between the controls of HGBS and LGBS disappeared when both populations were inoculated with *Xcc.* HGBS was significantly less damaged than LGBS. Taking this information into account, we suggest that high GBS content confers a defensive advantage that balances the investment in this defensive compound and the physiological status of the plant. Agreeing with this theory, the high content of SIN does not protect the plant against *Xcc*; therefore, LSIN was more resistant than HSIN and its physiological status was better. The same balance between growth and defense is observed in *Ss* experiment. HSIN performed better for physiological parameters than LSIN, probably due to their higher resistance to *Ss*. Agreeing with our results, [35] found an association between GSL content and increased seed production when predators were present in *A. thaliana*. However, the cost of this production was a fitness defect when predators were absent [36]. Employing a flux balance analysis, [15] found that GSL production can increase photosynthetic requirements by at least 15% in *A. thaliana*. Therefore, an improvement in GSL content has associated costs when plants are not subjected to an attack by pathogens, but costs are balanced by the plant under biotic stress. 

## 4. Materials and Methods

### 4.1. Pathogens and Plant Culture

A set of populations of *B. oleracea* var. *acephala* L. (kale) differing in the content of two GSLs, the aliphatic SIN (HSIN and LSIN) and the indolic GBS (HGBS and LGBS), were employed. The plant populations were obtained from a mass selection program at the Misión Biológica de Galicia, Spanish Council for Scientific Research (MBG-CSIC) [11]. For the experiments, populations were grown in a greenhouse with a 14 h photoperiod, a day–night mean temperature of 24/18 °C and 70% relative humidity. Plants were grown in pots containing 2.5 L of peat (Gramoflor GmbH & Co. KG Produktion, Vechta, Germany). 

The isolate of *Xcc* race (strain HRI3811, synonymous with PHW1205) was originally collected from *B. oleracea* in the United States and was provided by Joana Vicente (Warwick HRI, Wellesbourne, UK). The *Ss* isolate MBG-*Ss*2 was provided by MBG-CSIC. The original isolate was collected in January 2008 from a naturally infected plant of *Brassica napus* in an experimental field at MBG. To maintain pathogens against time, they were subcultured periodically in PDA medium. Cultures of *Xcc* were incubated at 30 °C and cultures of *Ss* were incubated with a 14 h photoperiod, a day–night mean temperature of 24/18 °C. 

### 4.2. Inoculation and Physiological Evaluation under Control and Biotic Stress Conditions

Two different experiments were performed. In the first one, plants were inoculated with *Xcc* and in the second one with *Ss*. The experimental design was the same in both experiments. One hundred and twenty plants from each genotype (HGBS, LGBS, HSIN, and LSIN) were employed. Since the progress of the disease varies considerable depending on the pathogen, plants inoculated with *Xcc* were monitored during four weeks and plants inoculated with *Ss* during five days. In both experiments, plants were divided into two sets of twenty plants each; one was inoculated with the pathogen and the other received no treatment. Plants within each treatment were arranged in three repetitions. 

One set of plants was inoculated and the other one received no treatment. In the first experiment, four weeks after sowing the plants, fresh bacterial colonies of *Xcc* were sub-cultured on Petri dishes containing PDA and incubated at 32 °C for 24 h in the dark. A loop of bacteria was transferred to nutrient broth and shaken overnight at 150 rpm and 30 °C in the dark. The culture was diluted in sterile water to a concentration of 5 × 10^8^ cfu·mL^−1^. Fresh bacterial inoculum was injected into three different points of each leaf. In the second experiment, fresh mycelium of *Ss* was obtained through routine transfer of mycelial–agar plugs from the margin of a colony of *Ss* growing on PDA medium. The cultures were incubated over a 14 h photoperiod, with a day/night mean temperature of 24/18 °C. Agar plugs containing the advancing edge of fungal mycelia were used as the inoculum source. Leaves were inoculated by placing one agar plug with the fungal inoculum on the upper side of the leaf. In both experiments, the second youngest leaf counting from the apex was inoculated with pathogen (inoculated plants) or with water (control plants). Inoculated leaves were photographed. Then, a lesion area was obtained using ImageJ software (Version 1.51n). 

The physiological status of the plants in both experiments was assessed using reflectance measurements and employing a UniSpec SC spectroradiometer (PP Systems, Haverhill, MA, USA). In both experiments, measurements were conducted in the inoculated leaf of each plant. Then, with the output matrix, several indexes were computed using AVICOL v.6 Software. Based on reflectance, different indexes have been proposed to check the physiological status of the plant and functional processes. In this work, we have employed indexes to estimate biomass, water and pigment content, and photosynthesis efficiency. Water content was estimated by employing WI900 or the ‘water index’. Biomass was estimated using CHL-NDVI or the ratio between reflectance at 750 and 550 nm (R750/R550) and NDVI index ‘normalized difference vegetation index’ and with SR or ‘simple ratio’ [16]. Pigment content was measured using SIPI, or the ‘structural independent pigment index’ that measures carotenoids/chlorophyll ratio and NPQI ‘normalized phaeophytinization index’ that measures the chlorophyll degradation. The YI ‘yellowness index’ [37] measures the chlorosis of leaves in stressed plants. RE or ‘red edge’ is an estimate of chlorophyll content. PR531 or the ‘photochemical radiation index’ indicates the photosynthetic radiation-use efficiency [16]. Chlorophyll fluorescence yield was measured in the same leaves as reflectance, using a portable Chlorophyll fluorometer (Mini-PAM; Heinz Walz, GmbH, Effeltrich, Germany). Fluorescent transience was induced using red light of 3000 μmoL m^−2^·s^−1^ provided by an array of 3 light-emitting diodes (peak at 660 nm) using plants dark-adapted for 20 min. Part of the light energy absorbed by chlorophyll for photosynthesis is lost as heat or fluorescence; thus, measurements in fluorescence emissions can be employed to assess chlorophyll efficiency. Then, we employed the F_v_F_m_ ratio based on fluorescence and the PR531 index based on infrared reflectance as a proxy of photosynthesis efficiency. 

### 4.3. Statistical Analysis

Analyses of variance were performed using the GLM procedure of Statistical Analysis Software (SAS, Institute Inc., Cary, NC, USA). An individual analysis of variance was computed by experiment (*Xcc*, *Ss*) and time. Comparisons of means between treatments were conducted using a Student’s t test at the 0.05 level of probability. Three different analyses were performed. First of all, we compared the physiological status between the controls of the high and low GSLs populations. Then, we compared the physiological status of the controls vs. inoculated populations. Finally, we compared the performance between populations with high and low GSL content inoculated with *Xcc* or *Ss*. Populations and treatments were considered fixed factors, whereas replications were considered random factors.

## 5. Conclusions

The modification of the constitutive content of GSLs has dual consequences for both the resistance to necrotrophic pathogens and also in several physiological aspects of plants. In this way, by increasing the content of sinigrin and glucobrassicin, plants decrease photosynthesis efficiency (PR531, F_v_F_m_), biomass production (CHL_NDVI, SR), pigment content (SIPI, NPQI, RE), and their level of senescence (YI) and increase their water content (WI900). These physiological changes have the potential to negatively impact crop productivity in agricultural environments. However, when plants with increased GSL content are exposed to necrotrophic pathogen attacks, the elevated levels of sinigrin and glucobrassicin can confer an adaptive advantage. This advantage compensates for the negative effects on physiological parameters, allowing the plants to better withstand and combat the pathogens. This study highlights the potential benefits of increased GSL content in mitigating pathogen-induced production losses. Further research is needed to evaluate the broader ecological and agronomic implications and to develop a comprehensive understanding of the long-term effects on crop productivity and sustainability.

## Supplementary Materials

The following supporting information can be downloaded at https://www.mdpi.com/article/10.3390/plants12142698/s1, Figure S1: Spectral reflectance in the visible and in the near-infrared of populations with high (H) and low (L) content of glucobrassicin (GBS), treated with *Xanthomonas campestris* pv. *campestris* (I) or with no treatment (C), 21 days post inoculation. Data are the average of 20 plants. Figure S2: Spectral reflectance in the visible and in the near-infrared of populations with high (H) and low (L) content of sinigrin (SIN), treated with *Xanthomonas campestris* pv. *campestris* (I) or with no treatment (C), 21 days post inoculation. Data are the average of 20 plants. Figure S3: Spectral reflectance in the visible and in the near-infrared wavelengths of populations with high (H) and low (L) content of glucobrassicin (GBS), treated with *Sclerotinia sclerotiorum* (I) or with no treatment (C), four days post inoculation. Data are the average of 20 plants. Figure S4: Spectral reflectance in the visible and in the near-infrared of populations with high (H) and low (L) content of sinigrin (SIN), treated with *Sclerotinia sclerotiorum* (I) or with no treatment (C), four days post inoculation. Data are the average of 20 plants.
