# Supplementary material for: Spectral Reflectance Indexes Reveal Differences in the Physiological Status of Brassica oleracea with Contrasting Glucosinolate Content under Biotic Stress
Source: Plants (Basel). 2023 Jul 19;12(14):2698. doi: 10.3390/plants12142698 (PMC10384497; doi:10.3390/plants12142698)

Figure S1. Spectral reflectance in the visible and in the near-infrared of populations with high (H) and low (L) content of glucobrassicin (GBS), treated with *Xanthomonas campestris* pv. *campestris* (I) or with no treatment (C), 21 days post inoculation. Data is the average of 20 plants.

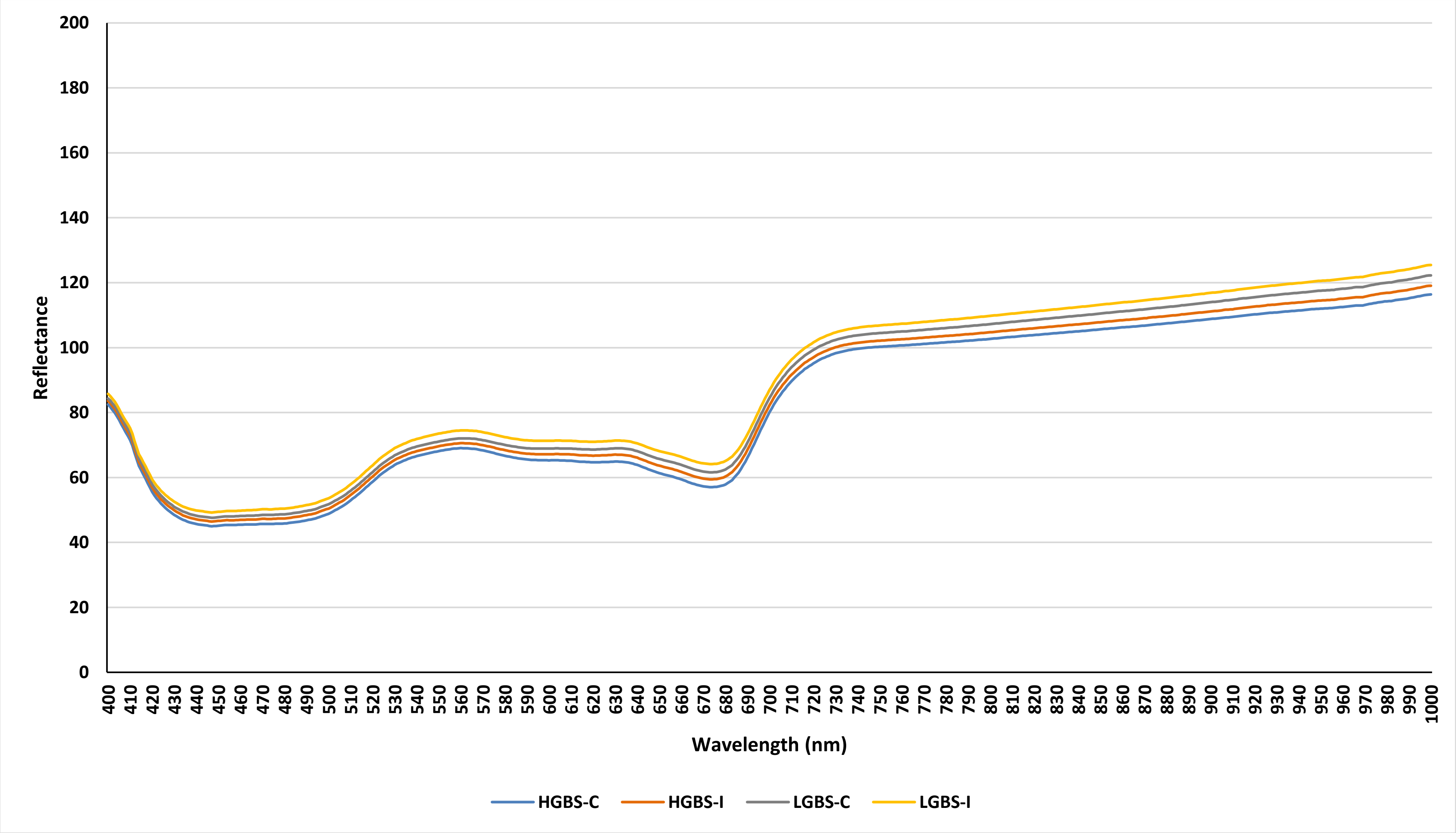

Figure S2. Spectral reflectance in the visible and in the near-infrared of populations with high (H) and low (L) content of sinigrin (SIN), treated with *Xanthomonas campestris* pv. *campestris* (I) or with no treatment (C), 21 days post inoculation. Data is the average of 20 plants

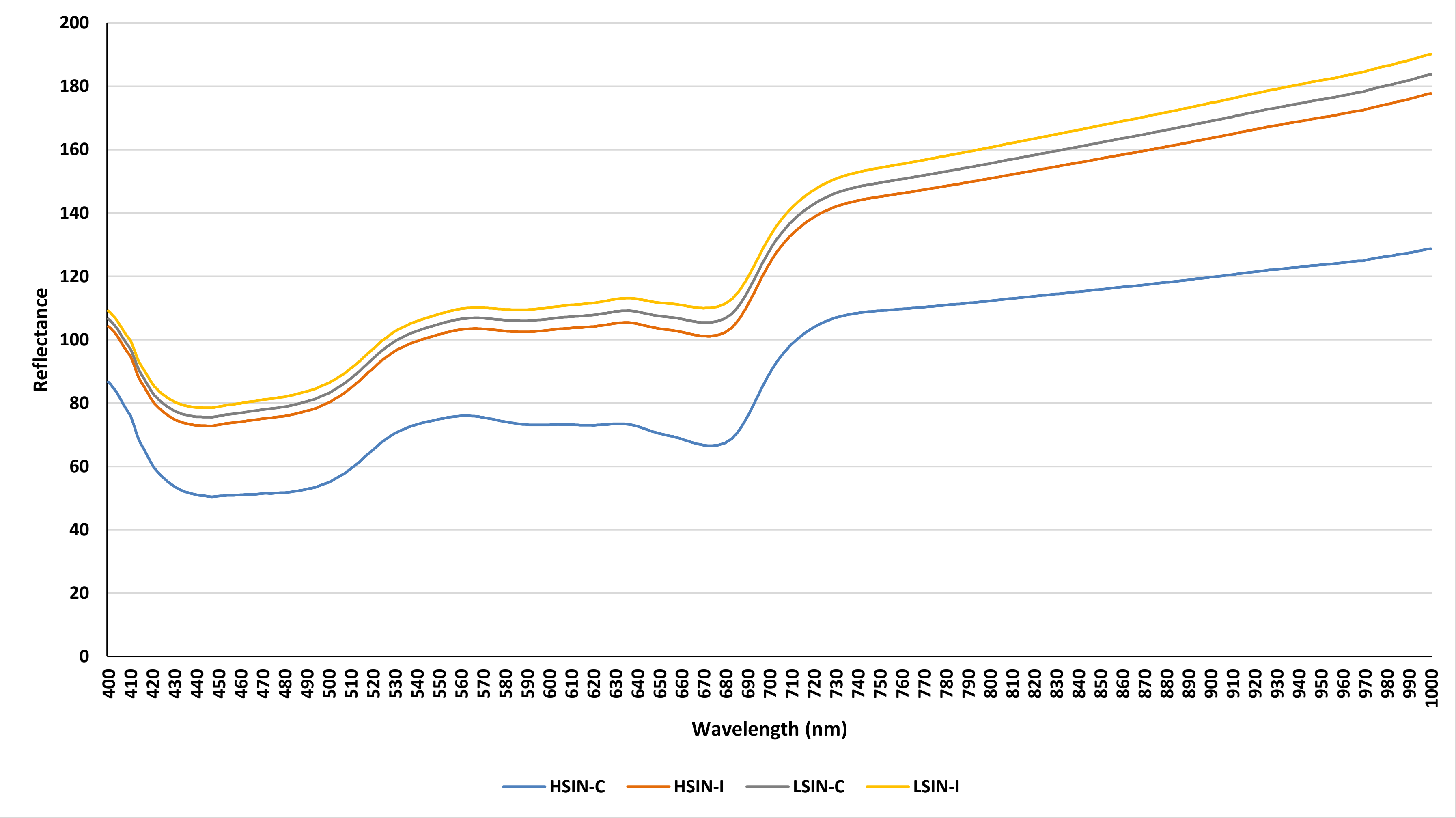

Figure S3. Spectral reflectance in the visible and in the near-infrared wavelegnths of populations with high (H) and low (L) content of glucobrassicin (GBS), treated with *Sclerotinia sclerotiorum* (I) or with no treatment (C), four days post inoculation. Data is the average of 20 plants

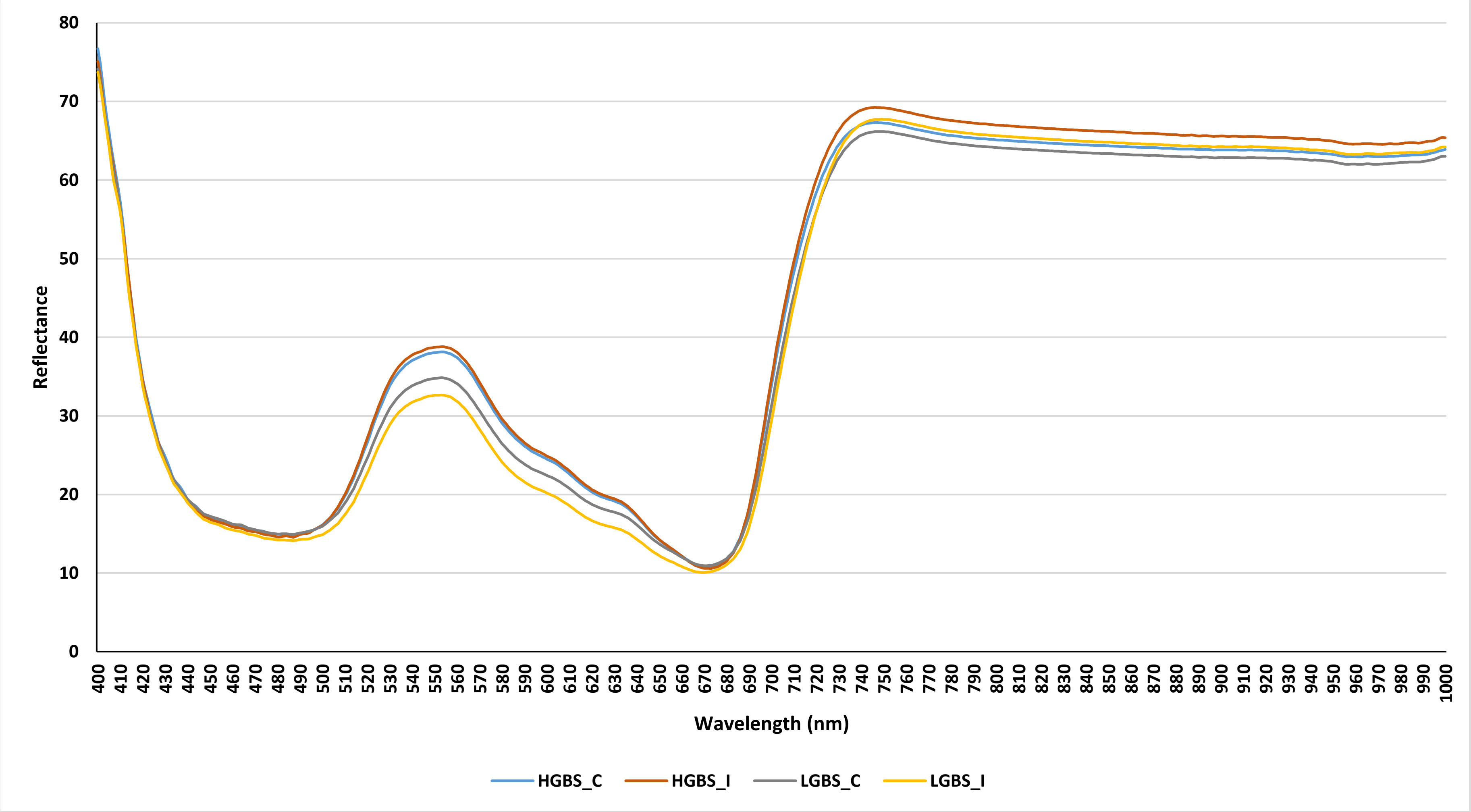

Figure S4. Spectral reflectance in the visible and in the near-infrared of populations with high (H) and low (L) content of sinigrin (SIN), treated with *Sclerotinia sclerotiorum* (I) or with no treatment (C), four days post inoculation. Data is the average of 20 plants

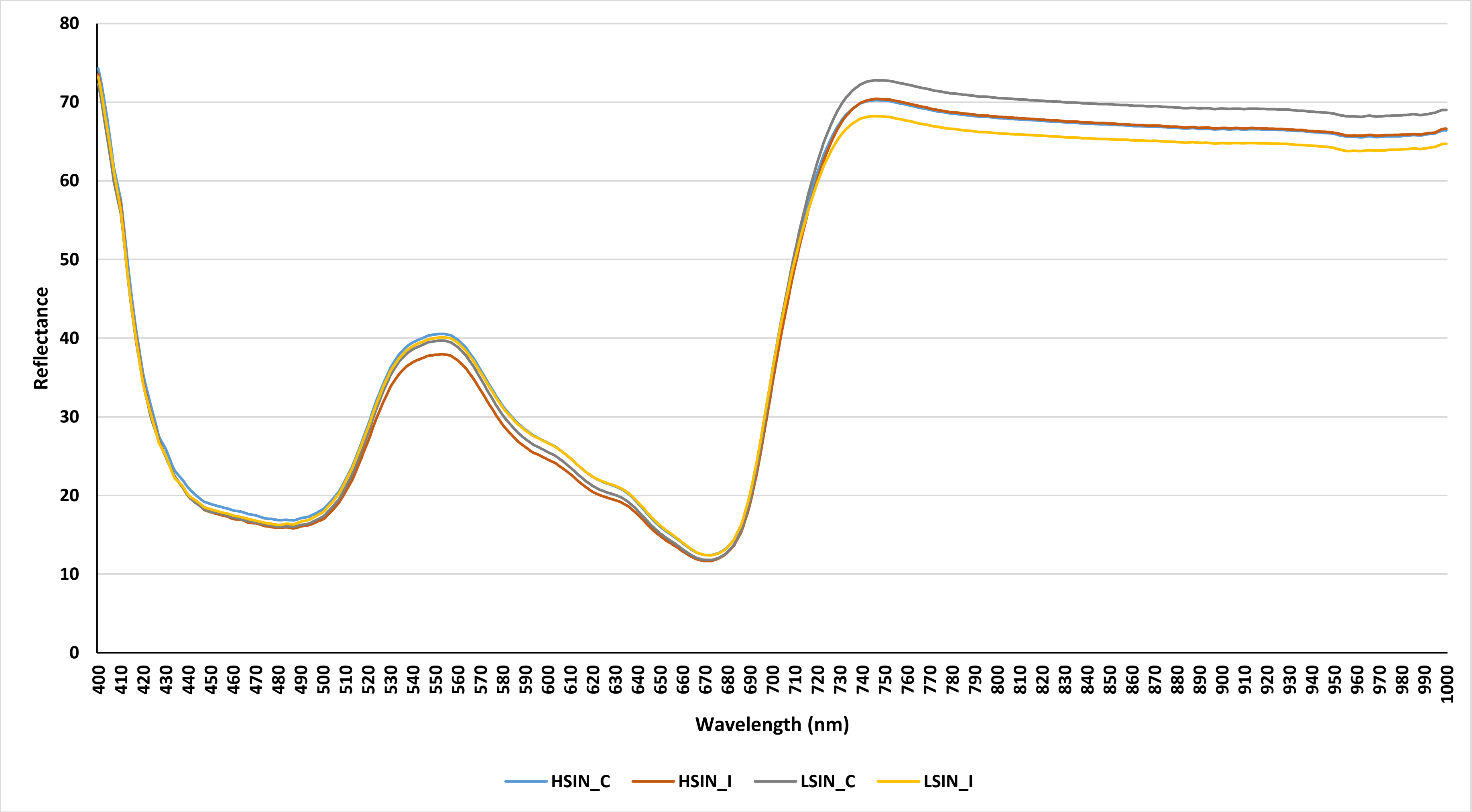

Supplement: Supplementary file 1 [file plants-12-02698-s001.zip › Supplementary Figures.pdf]
